# Nonclassical Spin‐Multiplexing Metasurfaces Enabled Multifunctional Meta‐Scope

**DOI:** 10.1002/smll.202404003

**Published:** 2024-09-23

**Authors:** Chuang Sun, Zixuan Wang, Kian Shen Kiang, Oleksandr Buchnev, Dawei Tang, Jize Yan, Jun‐Yu Ou

**Affiliations:** ^1^ School of Electronics and Computer Science University of Southampton Southampton SO17 1BJ UK; ^2^ Optoelectronics Research Centre University of Southampton Southampton SO17 1BJ UK; ^3^ Centre for Precision Technologies University of Huddersfield Huddersfield HD1 3DH UK; ^4^ School of Physics and Astronomy University of Southampton Southampton SO17 1BJ UK; ^5^ Institute for Life Sciences University of Southampton Southampton SO17 1BJ UK

**Keywords:** metalens, meta‐scope, metasurfaces, multiple foci, optical microscopy

## Abstract

Dielectric metasurfaces have emerged as attractive devices for advanced imaging systems because of their high efficiency, ability of wavefront manipulation, and lightweight. The classical spin‐multiplexing metasurfaces can only provide two orthogonal circular polarization channels and require high phase contrast which limits their applications. Here, metasurfaces with arbitrary three independent channels are demonstrated by proposing a nonclassical spin‐multiplexing approach exploring the low refractive index meta‐atoms. A zoom microscope with on‐axis tri‐foci and a synchronous achiral‐chiral microscope with in‐plane tri‐foci based on silicon nitride metasurfaces are experimentally demonstrated. Based on the on‐axis tri‐foci metasurface, singlet zoom imaging with three magnifications and a broadband response (blue to red) based on a single metasurface is first demonstrated. A compact microscope (meta‐scope) consisting of two metasurfaces with three magnifications of 9.5, 10, and 29X with diffraction‐limited resolutions is further constructed, respectively. Utilizing the in‐plane tri‐foci metasurface, a singlet microscope with three achiral‐chiral channels is demonstrated. It offers a magnification of 53X and a diffraction‐limited resolution, enabling simultaneous imaging of an object's achiral and chiral properties. Our multifunctional metasurfaces and meta‐scope approaches could boost the applications in biological imaging and machine vision.

## Graphical abstract

While the classical spin‐multiplexing dielectric metasurfaces require a high‐refraction‐index material and can only achieve two orthogonally circular polarisation channels, a nonclassical spin‐multiplexing approach is developed for achieving tri‐channel metasurfaces via a low‐refraction‐index material in this paper. In experiments, an inherent zoom meta‐scope and a simultaneously achiral and chiral meta‐scope are demonstrated.

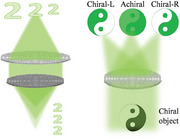

## 1 Introduction

Since the generalized Snell law and the abrupt phase control over the wavelength scale were reported in the year of 2011,^[^ ^1^ ^]^ the metasurface research has grown explosively around the world due to the metasurface's powerful ability of manipulation in amplitude,^[^ ^2^, ^3^ ^]^ phase,^[^ ^4^, ^5^ ^]^ polarization,^[^ ^6^, ^7^ ^]^ and even frequency^[^ ^8^, ^9^ ^]^ of electromagnetic waves. The all‐dielectric metasurface is attracting intensive attention as it can avoid ohmic losses and realize higher efficiency.^[^ ^10^ ^]^ The working mechanism of an all‐dielectric metasurface can be based on resonance effects (e.g., Mie‐resonance, Fano resonance, bound states in the continuum, etc.) or nonresonance effects (e.g., propagation phase, geometry phase, and their combination).^[^ ^10^, ^11^, ^12^, ^13^ ^]^ The imaging application of resonant phase focusing metalens would be restricted by its narrow bandwidth and the strong resonance coupling between adjacent meta‐atoms.^[^ ^14^ ^]^ In contrast, metalens based on nonresonant phases can work for tightly focusing with a high numerical aperture (NA) with a wide spectrum. For example, the metalens based on truncated waveguide meta‐atom has been adopted for high‐efficiency, high‐resolution, and broadband imaging in the ultraviolet, visible, and infrared spectrum.^[^ ^15^, ^16^, ^17^, ^18^, ^19^ ^]^

With the development of an anisotropic truncated meta‐atom, the classical spin‐multiplexing principle is developed and adopted for achieving multifunctional metasurfaces based on one set of meta‐atoms.^[^ ^13^, ^20^, ^21^ ^]^ For achieving spin‐multiplexing, the meta‐atoms require a *π* phase delay between the vertically and horizontally polarized light beams and a 2*π* phase variation of the propagation phase. Therefore, a high‐refraction index and low‐absorption material should be adopted for building spin‐multiplexing metasurfaces. Generally, silicon is used in near‐infrared, mid‐infrared, and terahertz spectrums, while Silicon Nitride (Si_3_N_4_), Titanium Dioxide (TiO_2_), and Gallium Nitride (GaN) are widely used in the visible spectrum.^[^ ^16^, ^17^, ^19^, ^20^, ^21^, ^22^, ^23^, ^24^ ^]^ However, the fabrication of TiO_2_ and GaN metasurfaces is not compatible with the silicon‐based complementary metal‐oxide‐semiconductor (CMOS) fabrication process and requires a complex process of low‐temperature atomic layer deposition and high aspect ratio two‐step dry etching,^[^ ^16^ ^]^ which would increase the fabrication costs of TiO_2_ and GaN metasurfaces and limit their monolithic integration with on‐chip photonics and electronics devices. In contrast, Si_3_N_4_ metasurfaces are CMOS‐compatible and broadband transparent. However, the multifunctional imaging application of Si_3_N_4_ metasurfaces was limited previously because of the low‐refraction index. In this paper, we exploit a nonclassical spin‐multiplexing approach (NSMA) for tri‐channel multifunctional metasurfaces via a low‐refraction‐index material (e.g., Si_3_N_4_). “Nonclassical” is adopted here to highlight the difference between our proposed approach and the wildly used spin‐multiplexing method which can only achieve two spin‐selective channels.

Meta‐scopes refer to a microscope system with one or two metalens that have demonstrated advantages over conventional microscopes. For example, one tunable varifocal metalens can realize zoom imaging^[^ ^25^, ^26^, ^27^, ^28^ ^]^ and the spin‐multiplexing metalens can integrate two imaging functions into one meta‐scope.^[^ ^21^, ^29^ ^]^ In previous studies, external control signals (e.g., electric and stress)^[^ ^25^, ^27^ ^]^ and phase transition material^[^ ^28^ ^]^ are required to adjust the varifocal metalens and to achieve zoom imaging application. The classical spin‐multiplexing metasurfaces could only achieve two independent functions limited by the two orthogonal left‐handed and right‐handed circular polarization (LCP and RCP) channels.^[^ ^29^, ^30^ ^]^ As a result, a classical spin‐multiplexing meta‐scope cannot realize simultaneously achiral and chiral (LCP and RCP) imaging.

**Figure 1:**
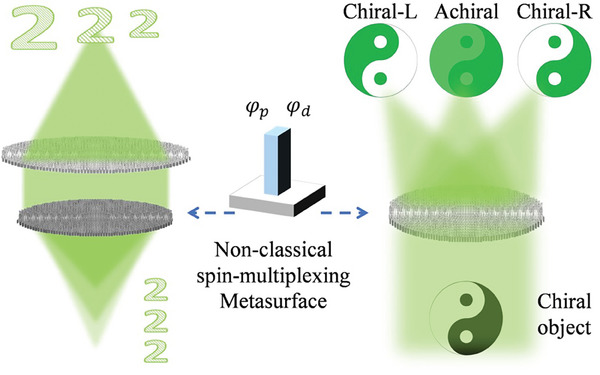
Concept of meta‐scope based on nonclassical spin‐multiplexing metasurface. Left, the zoom meta‐scope is based on the on‐axis tri‐foci metasurface. Right, the synchronous achiral‐chiral meta‐scope is based on an in‐plane tri‐foci metasurface.

Based on amorphous‐silicon (a‐Si), Rho's research group demonstrated the tri‐channel encryption meta‐displays^[^ ^31^ ^]^ by using the spin‐multiplexing metasurface and Malus’ law intensity modulation and achieved the tri‐channel spin‐selective metalens^[^ ^32^ ^]^ by combining the geometric and propagation phase. However, the a‐Si material has high absorption in the visible spectrum which limits visible imaging applications. By independently modulating the phase profiles of one co‐circular‐polarization and two cross‐circular‐polarization lights, a tri‐channel chiral metasurface was demonstrated to construct the high‐spatial‐resolution polarimetry based on Silicon Nitride, SiNx.^[^ ^33^ ^]^ However, high‐performance polarimetry is achieved by incorporating a machine‐learning approach. Therefore, it is hard to evaluate the metasurface's performance directly. In addition, except for the above classical‐regime applications, the multifunctional metalenses can also be used for quantum emission.^[^ ^34^ ^]^ In this paper, we experimentally demonstrated the inherent zoom microscope (meta‐scope) with an on‐axis tri‐foci metalens with three magnifications and the simultaneously achiral and chiral meta‐scope with an in‐plane tri‐foci metalens with diffraction‐limited resolution and large magnification of 53X (**Figure** **1**).

This paper is constructed as follows. In Section 2, the NSMA is proposed based on a general working principle of a meta‐atom. The on‐axis tri‐foci Si_3_N_4_ metalens and zoom meta‐scope are demonstrated in Section 3. Section 4 demonstrates the in‐plane tri‐foci Si_3_N_4_ metalens as well as the simultaneously achiral and chiral meta‐scope. Finally, a discussion and conclusion are presented in Section 5.

## 2 Nonclassical Spin‐Multiplexing Approach

For dielectric metalens based on the nonresonant meta‐atoms, the propagation phase can be explained by regarding the meta‐atom (Figure S1a, Supporting Information) as a truncated waveguide.^[^ ^4^ ^]^ Equation (1) illustrates the propagation phase φ_*p*_ induced by the meta‐atom, where *n_e_* is the effective refraction index, *H* is the height of the meta‐atom, and λ is the working wavelength. As the *n_eff_* is polarization dependent for an anisotropic meta‐atom, two distinct propagation phases (φ_*x*_ and φ_*y*_) could be imposed on the ongoing X‐ and Y‐polarized light, which results in the meta‐atom working as a waveplate and can be expressed by a Jones matrix $\left[\begin{array}{cc} t_{x}e^{i\varphi_{x}} & 0\\ 0 & t_{y}e^{i\varphi_{y}} \end{array}\right]$ where *t_x_* and*t_y_* are the transmission of X‐ and Y‐polarized light.

(1) $$\varphi_{p}=\frac{2\pi }{\lambda }\;n_{eff}H$$

(2) $$|E_{o}\rangle =\sqrt{\eta_{i}}e^{i\left(\varphi_{p}\right)}|E_{i}\rangle +\sqrt{\eta_{L}}e^{i\left(2\theta +\varphi_{p}\right)}|L\rangle +\sqrt{\eta_{R}}e^{e^{i\left(-2\theta +\varphi_{p}\right)}}|R\rangle$$

Arising from the scattering effect, as illustrated by Equation (2), the ongoing light|*E_o_* 〉 of a truncated waveguide, the meta‐atom has three polarization channels.^[^ ^4^ ^]^ The first channel has an identical polarization state with the incident light beam |*E_i_* 〉, and the second and third channels denote the left‐circularly polarized (LCP, |*L*〉) and right‐circularly polarized (RCP, |*R*〉) light.

In Equation (2), the $\eta_{i}={|\frac{1}{2}\left(t_{x}+t_{y}e^{i\varphi_{d}}\right)|}^{2}$, $\eta_{L}={|\frac{1}{2}\left(t_{x}-t_{y}e^{i\varphi_{d}}\right)\left\langle R|E_{i}\right\rangle |}^{2}$, $\eta_{R}={|\frac{1}{2}\left(t_{x}-t_{y}e^{i\varphi_{d}}\right)\left\langle L|E_{i}\right\rangle |}^{2}$  are the efficiency coupled to each channel, propagation phase $\varphi_{p}=\frac{\varphi_{y}+\varphi_{x}}{2}\;\approx \varphi_{x}$, phase delay φ_*d*_ = |φ_*y*_ − φ_*x*_|, and the rotation angle θ.^[^ ^4^ ^]^ Assuming that the *t_x_* and *t_y_* can be optimized to be higher than 90% in designing the meta‐atom, the three coupling efficiencies are simplified to $\eta_{i}={|\frac{1}{2}\left(1+e^{i\varphi_{d}}\right)|}^{2}$, $\eta_{L}={|\frac{1}{2}\left(1-e^{i\varphi_{d}}\right)\left\langle R|E_{i}\right\rangle |}^{2}$, and $\eta_{R}={|\frac{1}{2}\left(1-e^{i\varphi_{d}}\right)\left\langle L|E_{i}\right\rangle |}^{2}$ .

When φ_*d*_ =  0, η_*L*_ = η_*R*_  =  0, and η_*i*_ =  1, Equation (2) illustrates the polarization‐independent propagation phase for an isotropic meta‐atom. When φ_*d*_ =  π, η_*i*_ =  0, η_*L*_ and η_*R*_ rely on the polarization state of the incident light, Equation (2) illustrates the classical spin‐multiplexing meta‐atom. However, the regime of 0 < φ_*d*_ <  π has not been adopted to achieve Si_3_N_4_‐based tri‐channel metalenses and meta‐scope. We will discuss the advantage of low phase contrast of 0 < φ_*d*_ <  π in the following paragraphs.

We noted that when 0 < φ_*d*_ < π, three coupling efficiencies η_*i*_, η_*L*_, η_*R*_ are not zero when 〈*R*|*E_i_* 〉 ≠ 0 and 〈*L*|*E_i_* 〉 ≠ 0, which illustrates that a third channel exists in the ongoing light beam when the incident light beam is not perfect LCP and RCP. Each channel can be imposed on three different phase profiles. Φ_1_(*x*,*y*)  =  2θ(*x*, *y*) + φ_*p*_(*x*,*y*), Φ_3_ (*x*,*y*) =   − 2θ(*x*, *y*) + φ_*p*_(*x*,*y*), and 2Φ_2_ (*x*,*y*) = 2φ_*p*_  (*x*,*y*) = Φ_1_ (*x*,*y*) + Φ_3_(*x*,*y*). It means that the phase profile φ_*p*_ (*x*,*y*) = [Φ_1_ (*x*,*y*) + Φ_3_(*x*,*y*)]/2 applied to the polarization‐independent channel $\sqrt{\eta_{i}}e^{i(\varphi_{p})}\left|E_{i}\right\rangle$ is fully determined by two‐phase profiles of Φ_1_(*x*,*y*)  =  2θ(*x*, *y*) + φ_*p*_(*x*,*y*) and Φ_3_ (*x*,*y*) = − 2θ(*x*, *y*) + φ_*p*_(*x*,*y*) imposed on the LCP and RCP channels. When the incident light beam is perfect LCP (〈*R*|*E_i_* 〉 = 0) or RCP (〈*L*|*E_i_* 〉 = 0), the ongoing light beam would only consist of two channels. Therefore, when a metasurface works in the regime of 0 < φ_*d*_ < π, the ongoing light beams are spin‐dependent as well. Hence, in this paper, the design approach is based on a φ_*d*_ between 0 and π and is named as a nonclassical spin‐multiplexing approach (NSMA).

As we can build up a meta‐atom library where each meta‐atom possesses a fixed phase delay φ_*d*_ (0 < φ_*d*_ < π) and the propagation phase φ_*p*_ of all meta‐atoms can achieve 2π phase coverage, a tri‐channel metasurface could be achieved by applying three‐phase profiles [Φ_1_(*x*,*y*), Φ_2_(*x*,*y*), and Φ_3_(*x*,*y*)] on the ongoing light beam. The relaxed requirement for phase delay φ_*d*_ is smaller than π which enables the low‐refraction‐index material for tri‐channel metasurfaces based on the NSMA. The plasma‐enhanced chemical vapor deposited Si_3_N_4_ film with a refraction index of 2 was adopted for tri‐channel metasurfaces illustrating the merit of NSMA in the following proof‐of‐concept experiments.

As shown in supporting Figure S1b,c (Supporting Information), by fixing a period P of 350 nm and a height H of 800 nm for achieving two transmissions (*t_x_* and *t_y_*) higher 90%, the dependence of the propagation phase on the width W and length L of the meta‐atom was investigated in the simulation where the working wavelength is set as 520 nm. According to the phase maps in Figure S1c (Supporting Information), 14 meta‐atoms were selected for realizing 2π coverage of the propagation phase φ_*p*_ by fixing a phase delay φ_*d*_ of 5π/12. As a result, 63% (η_*i*_ =  63%) of the ongoing light energy could be coupled to the polarization‐independent achiral channel, and 37% (η_*L*_ +  η_*R*_ =  37%) of light energy would be coupled to the two chiral LCP and RCP channels. In the following Sections (3) and (4), two metalens samples and two tri‐channel meta‐scopes were demonstrated based on the NSMA and the meta‐atom library.

## 3 On‐Axis Tri‐Foci Metalens for Zoom Imaging Microscope

**Figure 2:**
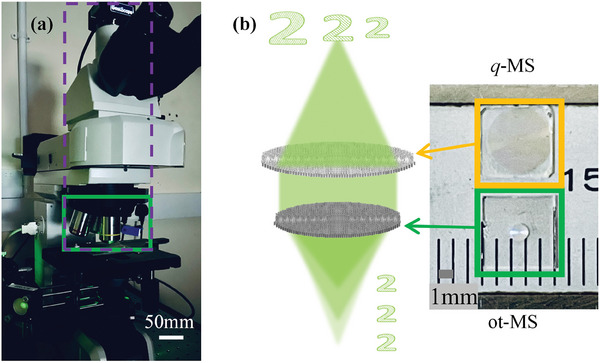
Conceptual figure of zoom imaging meta‐scope with three magnifications. a) Conventional infinity‐corrected microscope; b) shows the optical image of the on‐axis tri‐foci metalens (ot‐MS) and the quadratic phase metalens (q‐MS) which are used to build the zoom meta‐scope, scale bar 1 mm.

In a conventional microscope (**Figure** **2**a), an objective lens can provide only a single magnification.^[^ ^35^ ^]^ Multiple objective lenses are necessary for achieving different magnifications and resolutions, and a mechanical nosepiece is required to change the objective lenses (the section marked by the green rectangle in Figure 2a). As a result, the conventional microscope is bulky and heavy. According to the principle of an infinity‐corrected microscope, an on‐axis tri‐foci metalens can enable the monolithic integration of three objective lenses and a nosepiece (Figure 2b).

### 3.1 On‐Axis Tri‐Foci Metalens Design and Characterization

**Figure 3:**
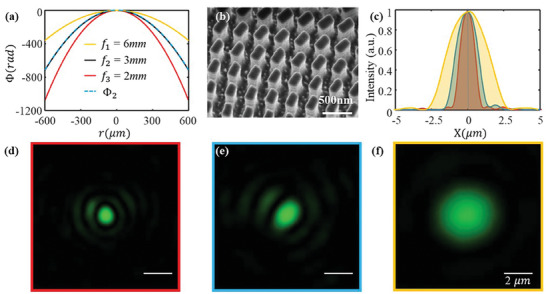
Design and optical characterization of the on‐axis tri‐foci metalens. a) Three hyperbolic phase profiles for the three focal points; b) SEM image of on‐axis tri‐foci metalens; c) the FWHMs of three focal points; d–f) are the three focal points at focal length *f*  =  1.9, 3, and 6 mm, respectively.

To achieve the on‐axis tri‐foci metalens, the phase profile Φ_1_(*x*,*y*) was designed as the hyperbolic phase profile Equation (3)^[^ ^17^ ^]^ with a focal length *f*_1_ of 6 mm, the phase profile Φ_3_(*x*,*y*) is the hyperbolic phase profile with a focal length *f*_3_ of 2 mm. Then, we can obtain the 2Φ_2_ (*x*,*y*) = Φ_1_ (*x*,*y*) + Φ_3_(*x*,*y*). According to Equation (4), the phase profile Φ_2_(*x*,*y*) is a hyperbolic phase with a focal length $f_{2}=\frac{2f_{1}f_{3}}{f_{1}+f_{3}}\;=\;3\;\mathrm{mm}.$ The three phase profiles are plotted in **Figure** **3**a in yellow, black, and red colors, respectively. In addition, from the black curve and blue dashed curve, the Φ_2_(*x*,*y*) agreed well with the hyperbolic phase profile Ψ( *f*_2_ =  3mm).

(3) $$\Psi \left(f,x,y\right)=-\frac{2\pi }{\lambda }\left(\sqrt{f^{2}+x^{2}+y^{2}}-f\right)$$

(4) $$2\Psi \left(\frac{2f_{1}f_{3}}{f_{3}+f_{3}},x,y\right)\approx \Psi \left(f_{1},x,y\right)+\Psi \left(f_{3},x,y\right)$$

Figures 2b and **3**b show the optical image and scanning electron microscope (SEM) image of the 1.2 mm‐diameter on‐axis tri‐foci metalens, respectively. Then, the on‐axis tri‐foci metalens was characterized by a home‐built microscope (see Section S2, Supporting Information). Section S3 (Supporting Information) illustrates the fabrication processes. We observed the first focal point (Figure 3d) by moving the imaging part of the microscope 1.9 mm away from the front surface of the on‐axis tri‐foci metalens. It means the first measured focal point has a focal length of 1.9 mm. With the moving microscope's imaging part to 3 and 6 mm away from the sample surface, we respectively observed the second (Figure 3e) and third focal points (Figure 3f). Therefore, three on‐axis focal points with the expected focal length are obtained. The NAs of three focal points can be calculated as 0.3 (*f*  =  1.9 mm), 0.2 (*f*  =  3 mm), and 0.1 (*f*  =  6 mm), respectively.

The focusing performance of the three focal points was evaluated by calculating their full‐width‐of‐half‐maximum (FWHM) (Figure 3c). The FWHMs of the three focal points are 1.18, 1.79, and 3.51 µm, respectively. The diffraction limitations corresponding to each focal point can be theoretically obtained as 1.02 µm (*f*  =  1.9 mm, NA = 0.3), 1.56 µm (*f*  =  3 mm, NA = 0.2), and 3.12 µm (*f*  =  6 mm, NA = 0.1). While the FWHMs are slightly larger than the diffraction limitations of each focal point, it can be concluded that three near‐diffraction‐limitation focal points are obtained via the on‐axis tri‐foci metalens. The wavefront errors arising from fabrication errors (Figure 3b) may be attributed to the nonperfect diffraction‐limited focusing performance (e.g., the outer rings in Figure 3d,e are slightly crushed). In addition, the focal point shown in Figure 3e has the same polarization with the incident laser beam and is elongated along the linearly polarized direction.^[^ ^36^ ^]^

### 3.2 Extending a Conventional Microscope for Zoom Imaging

**Figure 4:**
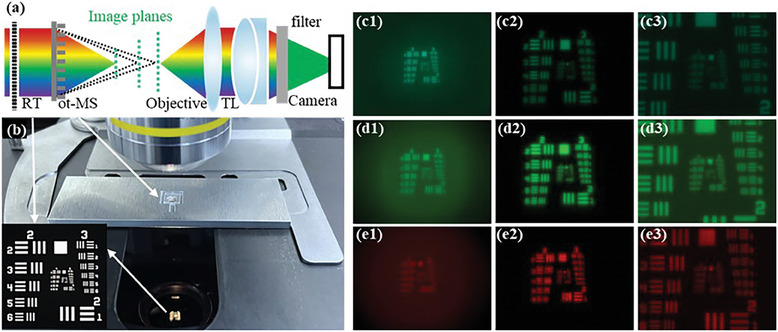
Singlet imaging experiment in the visible spectrum. a) Optical configuration. RT: resolution target [USAF 1951, see the insert in (b)]; TL: tube lens; b) Experiment setup; c–e) are the imaging results (the 1st column is obtained at *f*  =  1.9 mm, the 2nd column at *f*  =  3 mm, the 3rd column at *f*  =  6 mm) for the wavelength of 488, 520, and 650 nm, respectively. Insert figure in (b) is the ground truth of the RT.

The on‐axis tri‐foci metalens sample was first introduced to a conventional microscope (Figure 2a) for realizing zoom imaging based on an objective. As shown in **Figure** **4**a,b, a positive USAF 1951 resolution target was placed in the collimated illumination light path and was illuminated by a lamp with a wide emission spectrum. Passing through the on‐axis tri‐foci metalens, three images (Figure 4a) corresponding to three focal points can be obtained. Then, the three images were collected and magnified by the conventional microscope with an objective lens (10X, NA = 0.3). Because the chromatic aberration is not corrected in designing the on‐axis tri‐foci metalens, the resolution test target cannot be imaged to the same plane for different wavelengths. Therefore, a tunable bandpass filter (Thorlabs: KURIOS‐WB1/M) was placed in front of the camera to obtain images with a narrow bandwidth.

By setting the central wavelength of the filter at 488, 520, and 650 nm, we obtained nine images as shown in Figure 4c–e where a larger field of view (FOV) illustrates a smaller magnification. The images on Figure 4d1–d3 are respectively obtained at the 1^st^ (*f* = 1.9 mm), 2nd (*f* = 3 mm), 3rd (*f* = 6 mm) focal point. We can see that, the imaging FOVs get smaller with moving the objective lens from the 1st focal point to the 3rd one (Figure 4a), because the magnification of a singlet imaging system goes up with the increment of focal length for a fixed object distance (i.e., the distance between the resolution target and on‐axis tri‐foci metalens).

Comparing Figure 4c,e, the imaging FOV gets larger with tuning the working wavelength from 488 to 650 nm, which indicates that the magnification gets smaller with increasing the working wavelength. This phenomenon can be explained by the dispersion relation (i.e., the focal length gets smaller with increasing the working wavelength) of the adopted hyperbolic phase profile (Equation 3). Our results demonstrate that the on‐axis tri‐foci metalens can extend a conventional microscope to realize zoom imaging with a fixed objective in a wide spectrum range (488–650 nm).

### 3.3 Meta‐Scope for Zoom Imaging

**Figure 5:**
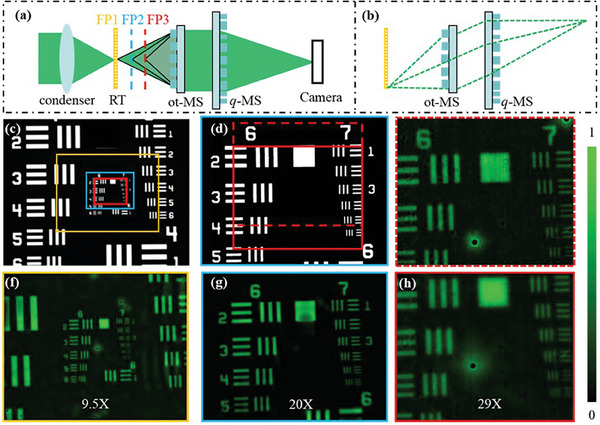
Compact meta‐scope experiment for zoom imaging. a) experimental configuration; b) optical path with off‐axis light trace; c,d) indicate the imaged FOV for each focal plane; f–h) are the captured images when the resolution target is respectively placed at *f*  = 6, 3, and 1.9 mm; e) is the second image captured at *f*  = 1.9 mm.

In the following, a meta‐scope that can be used for zooming imaging with three magnifications is demonstrated by using the on‐axis tri‐foci metalens as an objective lens. To miniaturize the whole meta‐scope to be centimeter level and lightweight, we designed and fabricated another metalens sample which was used a tube lens as shown in Figures 2b and **5**a. To realize a large FOV with low distortion, a quadratic phase profile Equation (5) was adopted in designing the second metalens sample (see Section S4, Supporting Information for details).^[^ ^37^ ^]^ Therefore, the second metalens is called quadratic phase metalens (Figures 2 and 5). In addition, the quadratic phase metalens has a diameter of 4.5 mm to increase the brightness of the image edge. As shown in Figure 5b, to effectively collect the light from an off‐axis object point, the quadratic phase metalens must have a larger diameter than the on‐axis tri‐foci metalens.^[^ ^35^ ^]^ Otherwise, the edge light energy cannot arrive at the camera. The focal length of the quadratic phase metalens is 45 mm for achieving high magnifications.

(5) $$\Psi_{q}\;\left(f,x,y\right)=\;-\frac{\pi \left(x^{2}+y^{2}\right)}{\lambda f}$$

As shown in Figure 5a, in the meta‐scope, the collimated light from a 520 nm LED was focused onto the resolution target via a condenser lens. The condenser lens and resolution target were mounted together so that they can be moved from focal plane 1 (FP1) to focal plane 3 (FP3) at the same time. As the compact meta‐scope is in an infinity‐corrected configuration, once the resolution target is placed in the three focal planes, the resolution target can be imaged to the focal plane of the quadratic phase metalens. Therefore, a camera is placed at the quadratic phase metalens focal plane to capture images.

Figure 5f–h illustrates the captured images when the resolution target is placed at *f*  = 6 mm (focal plane 1 marked in yellow in Figure 5a), 3 mm (focal plane 2 marked in blue in Figure 5a), and 1.9 mm (focal plane 3 marked in red in Figure 5a), respectively, and the measured FOVs are limited by the camera's sensor size (6.4 mm  ×  5.12 mm). The imaged regions as well as the FOVs of each focal plane are marked in yellow, blue, and red on a ground truth image (Figure 5c). As the red FOV (674 µm  ×  539 µm) is smaller than the blue one (320 µm  ×  256 µm) which is smaller than the yellow one (221 µm  ×  177 µm), the magnification gets larger with moving the resolution target from *f*  = 6 mm focal plane (Figure 5f) to *f*  = 1.9 mm focal plane (Figure 5h). The magnifications for Figure 5f–h are respectively 9.5, 20, and 29X based on quantitative calculation. When the resolution target is placed in the focal plane 3, as indicated by Figure 5d, we displaced the imaging region from the red solid rectangle to the red dashed rectangle to confirm the specific position (i.e., the group and element number). From Figure 5f, element 5 in group 7 of the resolution target can be clearly observed, which indicates that the meta‐scope can realize a resolution of 203.2 lp mm^−1^ at the focal plane 1 (focal length = 6 mm, NA = 0.1). Considering the other two focal points have diffraction‐limited FWHM (Figure 3e) as well, we claim that the meta‐scope can achieve diffraction‐limited resolutions (322.5 and 456 lp mm^−1^) at the focal planes 2 and 3.

## 4 In‐Plane Tri‐Foci Metalens for Simultaneously Achiral and Chiral Meta‐Scoping

Chiral imaging as an effective and fast way of detecting and identifying the chirality of biochemical objects has attracted increasing attention in biochemical and optical imaging research.^[^ ^38^, ^39^, ^40^ ^]^ However, there is no report on the tri‐channel metascope for simultaneously chiral and achiral imaging. Benefiting from the inherently three channels Equation (2) of the NSMA, we can obtain the in‐plane tri‐foci metalens for simultaneously achiral and chiral meta‐scoping.

### 4.1 In‐Plane Tri‐Foci Metalens Design and Characterization

**Figure 6:**
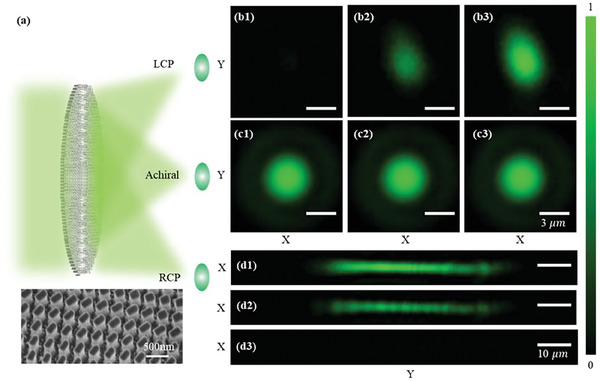
Concept and characterization of the in‐plane tri‐foci metalens for simultaneously chiral and achiral imaging. a) Conceptual figures; (b1–b3) as well as (d1–d3) are the measured chiral (LCP and RCP) focal points with tuning the laser beam from LCP to LP and to RCP; (c1)–(c3) are the achiral focal points. Scale bars in (b1)–(c3) are 3 µm, and scale bars in (d1)–(d3) are 10 µm.

As shown in **Figure** **6**a, the propagation phase term Φ_2_ (*x*,*y*) = φ_*p*_  (*x*,*y*) =  [Φ_1_(*x*,*y*) + Φ_3_(*x*,*y*)]/2 is imposed a hyperbolic profile with a focal length of 6 mm for achieving an on‐axis achiral focal point. The phase term Φ_3_ (*x*,*y*) =   − 2θ(*x*, *y*) + φ_*p*_(*x*,*y*) corresponding to the LCP channel is encoded an off‐axis hyperbolic phase profile Φ_*off*_ Equation (6) where α is the azimuthal angle corresponding to each nanopillar's position, β is the tilt angle of the LCP focal point,^[^ ^24^ ^]^ and *f*  =  6 mm. As a result, a phase profile Φ_1_ =  2Φ_2_ − Φ_3_ is encoded to the RCP channel. As discussed in Section S5 (Supporting Information), Φ_1_ is corresponding to an off‐axis focusing phase profile with a focal length of *f*  =  6 mm and a tilt angle of − β. Therefore, the distance from two off‐axis chiral focal points to the on‐axis achiral focal point is the same and equal to |*f* × *tan*(β)|. In the experiment, β is set to be 10^○^ to obtain a large separation distance *d* = 1.05 mm and avoid the overlap of achiral and chiral images. An in‐plane tri‐foci metalens sample with a diameter of 1.2 mm is fabricated following the process illustrated in Section S3 (Supporting Information).

(6) $$\Phi_{3}=\Phi_{off}\;\left(f,\beta \right)\;=\frac{2\pi }{\lambda }\;\left[f-\sqrt{f^{2}+r^{2}-2frsin\left(\beta \right)\cos\left(\alpha \right)}\right]$$

As shown in Figure 6b, we obtained three focal points at the target focal plane which is 6 mm away from the in‐plane tri‐foci metalens sample. With tuning the laser polarization state from being LCP to linear polarisation (LP) and to RCP, the LCP focal point's intensity increases to a maximum (Figure 6b1–b3), and the RCP focal point's intensity decreases to a minimum from a maximum value (Figure 6d1–d3), which demonstrates the chirality of two focal points. In addition, the on‐axis achiral focal point (Figure 6c1–c3) doesn't change with tuning the incident laser's polarization state.

The FWHM values of the achiral focal point are measured to be 3.2 µm which corresponds to the diffraction limitation of the 0.1 NA. In addition, the FWHM values of two chiral focal points are 3.2 µm as well along the X‐direction. As two chiral focal points are formed via the inclined focusing in y‐axis, the FWHM values of two chiral focal points are larger than 3.2 µm in the Y‐direction. Especially, the phase difference Φ_1*d*_ between the Φ_1_ and Φ_*off*_(*f*  = 6 mm, β  =   − 10°) results in that the RCP cannot be perfectly focused in the Y‐direction (see Section S5, Supporting Information for details). Based on this metalens, a simultaneously chiral and achiral meta‐scope system is demonstrated in the following.

### 4.2 Singlet Meta‐Scope for Simultaneously Chiral and Achiral Imaging

**Figure 7:**
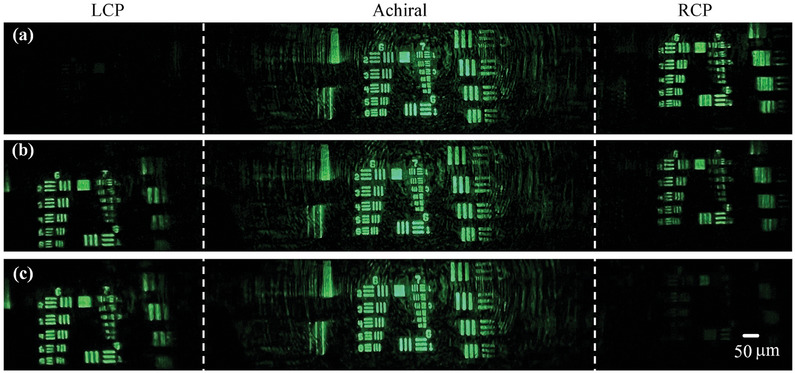
Singlet meta‐scope for simultaneously chiral and achiral imaging. a–c) The illumination laser beam is RCP, LP, and LCP, respectively.

As shown in Section S6 (Supporting Information), the meta‐scope system is in a transmissive configuration, and a USFA 1951 resolution target is illuminated by a focused 520 nm laser beam. The laser beam passing through the resolution target was collected by the in‐plane tri‐foci metalens sample and imaged on a whiteboard. The resolution target was placed 6.11 mm away from the front surface of the in‐plane tri‐foci metalens sample (i.e., the object distance equals 6.11 mm), and a clear image can be obtained when the whiteboard is placed 325 mm away from the in‐plane tri‐foci metalens. The meta‐scope system is illustrated in Figure S7 (Supporting Information). An iPhone 12 camera was adopted to capture clear images on the whiteboard (Figure S7b). The magnification of this meta‐scope system is calibrated by placing a ruler in front of the whiteboard. According to the ruler's dimension and the dimension of the resolution target in the captured image (Figure S7c, Supporting Information), the meta‐scope has a magnification of 53 times and a resolution of 3.1 µm ((i.e., 161.3lp/mm, element 3 in group 7, Figure S7d, Supporting Information). **Figure** **7** demonstrates the captured images by tuning the illumination laser beam's polarization.

As shown in Figure 7, by tuning the illumination light beam's polarization state from being RCP (Figure 7a) to LP (Figure 7b) to LCP (Figure 7c), the image corresponding to the LCP channel gradually gets brightest from dark, and the brightest image corresponding to the RCP channel gradually becomes dark, which demonstrates that two chiral imaging channels are achieved. Moreover, the image corresponding (the middle column in Figure 7a–c) to the on‐axis channel keeps steady with changing the polarization state, which shows that the on‐axis channel is achiral. Video S1 (Supporting Information) shows the transition process by rotating the quarter‐waveplate. These results demonstrate that a simultaneously chiral and achiral meta‐scope with a high magnification of 53 times and diffraction‐limited resolution is achieved.

**Figure 8:**
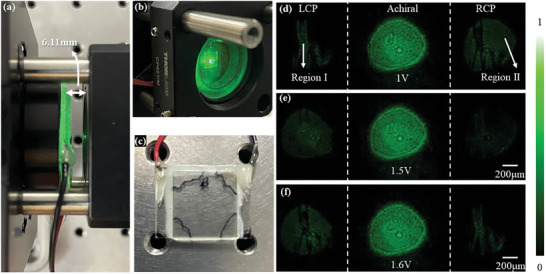
Fast detecting the chirality of an LC sample. a,b) singlet meta‐scope setup, c) transparent liquid crystal sample with defects, d–f) are the images with tuning the electrical voltage.

Lastly, the resolution target was replaced by a liquid crystal sample (see Section S7, Supporting Information for the preparation detail of this sample) to test the singlet meta‐scope system's ability to detect the chirality of a chemical sample, as shown in **Figure** **8**a–c. The liquid crystal sample is totally transparent in the visible spectrum as shown in Figure 8c, while the fast‐axis and slow‐axis of the LC cells in one region (i.e., Region I) are opposite to the other region (i.e., Region II). It means that the chirality of Region I would be opposite to that Region II when a quarter‐wave voltage is applied. In addition, when a half‐wave voltage is applied, the whole liquid crystal sample works as a half‐waveplate.

As shown in Figure 8d–f, the achiral images keep a uniform intensity pattern with changing the load voltage. In contrast, we obtained complementary images in the LCP and RCP channels, when the quarter‐wave voltage of 1 V is applied. Specifically, as shown in Figure 8d, the LCP channel obtains a bright image of Region I, and the image of Region I obtained by the RCP channel is dark. With increasing the loading voltage to a half‐wave voltage of 1.5 V, three chiral and achiral channels obtain almost the same pattern (Figure 8e). Continuously increasing the loading voltage to the second quarter‐wave voltage of 1.6 V, the LCP and RCP channels respectively obtain a dark and bright image of Region I (Figure 8f). This result demonstrates the fast detection ability of chirality transition with increasing the loading voltage of the liquid crystal sample (See Video S2, Supporting Information for the dynamical process).

## 5 Conclusion

In conclusion, a nonclassical spin‐multiplexing approach is proposed for tri‐channel metasurfaces via a low‐refraction‐index dielectric material. We experimentally demonstrated an on‐axis tri‐foci Si_3_N_4_ metalens and built a compact zoom meta‐scope for imaging with three large magnifications and diffraction‐limited resolutions. In addition, an in‐plane tri‐foci Si_3_N_4_ metalens with a 0.1 NA and a singlet meta‐scope were also experimentally demonstrated to achieve simultaneously chiral and achiral meta‐scope with a high magnification of 53 times and a diffraction‐limited resolution of 3.1 µm, which was validated by a liquid crystal sample. We conceive this research would boost the machine vision industry and biological imaging applications of meta‐scopes as well as the utilization of low‐refraction‐index dielectric metalenses.

## Conflict of Interest

The authors declare no conflict of interest.

## Supporting information



Supporting Information

Supplemental Video 1

Supplemental Video 2

## Data Availability

All data needed to evaluate the conclusions in the paper are present in the paper and the Supplementary Materials.

## References

[1] N. Yu , P. Genevet , M. A. Kats , F. Aieta , J.‐P. Tetienne , F. Capasso , Z. Gaburro , Science 2011, 334, 333. 21885733 10.1126/science.1210713

[2] S.‐J. Kim , I. Kim , S. Choi , H. Yoon , C. Kim , Y. Lee , C. Choi , J. Son , Y. W. Lee , J. Rho , B. Lee , Nanoscale Horiz. 2020, 5, 1088. 32377648 10.1039/d0nh00139b

[3] J. Liao , S. Guo , L. Yuan , C. Ji , C. Huang , X. Luo , Adv. Opt. Mater. 2022, 10, 2101551.

[4] D. Lin , P. Fan , E. Hasman , M. L. Brongersma , Science 2014, 345, 298. 25035488 10.1126/science.1253213

[5] R. C. Devlin , A. Ambrosio , N. A. Rubin , J. P. B. Mueller , F. Capasso , Science 2017, 358, 896. 29097490 10.1126/science.aao5392

[6] A. H. Dorrah , N. A. Rubin , A. Zaidi , M. Tamagnone , F. Capasso , Nat. Photonics 2021, 15, 287. 10.1038/s41467-021-26253-4 PMC8556329 34716326

[7] Y. Intaravanne , X. Chen , Nanophotonics 2020, 9, 1003.

[8] J. Zhang , X. Wei , I. D. Rukhlenko , H.‐T. Chen , W. Zhu , ACS Photonics 2019, 7, 265.

[9] J. C. Ke , J. Y. Dai , J. W. Zhang , Z. Chen , M. Z. Chen , Y. Lu , L. Zhang , L. Wang , Q. Y. Zhou , L. Li , J. S. Ding , Q. Cheng , T. J. Cui , Light: Sci. Appl. 2022, 11, 273. 36104318 10.1038/s41377-022-00973-8 PMC9474547

[10] T. Chung , H. Wang , H. Cai , Nanotechnology 2023, 34, 402001. 10.1088/1361-6528/ace117 PMC10416613 37352839

[11] D. Jeon , K. Shin , S.‐W. Moon , J. Rho , Nano Converg. 2023, 10, 24. 37222959 10.1186/s40580-023-00372-8 PMC10209387

[12] M. K. Chen , Y. Wu , L. Feng , Q. Fan , M. Lu , T. Xu , D. P. Tsai , Adv. Opt. Mater. 2021, 9, 2001414.

[13] A. Arbabi , A. Faraon , Nat. Photonics 2023, 17, 16.

[14] M. Pan , Y. Fu , M. Zheng , H. Chen , Y. Zang , H. Duan , Q. Li , M. Qiu , Y. Hu, Light Sci. Appl. 2022, 11, 195. 35764608 10.1038/s41377-022-00885-7 PMC9240015

[15] Y. Zhu , G. Yuan , Y. Chang , S. Zhou , C. Wu , Y. Li , W. Liu , Results Phys. 2023, 50, 106591.

[16] S. Colburn , A. Zhan , E. Bayati , J. Whitehead , A. Ryou , L. Huang , A. Majumdar , Opt. Mater. Express 2018, 8, 2330.

[17] Z.‐B. Fan , Z.‐K. Shao , M.‐Y. Xie , X.‐N. Pang , W.‐S. Ruan , F.‐L. Zhao , Y.‐J. Chen , S.‐Y. Yu , J.‐W. Dong , Phys. Rev. Appl. 2018, 10, 014005

[18] D. Zhao , Z. Lin , W. Zhu , H. J. Lezec , T. Xu , A. Agrawal , C. Zhang , K. Huang , Nanophotonics 2021, 10, 2283.

[19] Z.‐B. Fan , H. ‐Y. Qiu , H.‐L. Zhang , X.‐N. Pang , L.‐D. Zhou , L. Liu , H. Ren , Q.‐H. Wang , J.‐W. Dong , Light Sci. Appl. 2019, 8, 67. 31666943 10.1038/s41377-019-0178-2 PMC6804934

[20] H. Ren , G. Briere , X. Fang , P. Ni , R. Sawant , S. Héron , S. Chenot , S. Vézian , B. Damilano , V. Brändli , S. A. Maier , P. Genevet , Nat. Commun. 2019, 10, 2986. 31324755 10.1038/s41467-019-11030-1 PMC6642184

[21] P. Huo , C. Zhang , W. Zhu , M. Liu , S. Zhang , S. Zhang , L. Chen , H. J. Lezec , A. Agrawal , Y. Lu , T. Xu , Nano Lett. 2020, 20, 2791. 32155076 10.1021/acs.nanolett.0c00471 PMC7547647

[22] L. Yu , Y. Fan , Y. Wang , C. Zhang , W. Yang , Q. Song , S. Xiao , Laser Photonics Rev. 2020, 14, 1900324.

[23] Z. Li , C. Chen , Z. Guan , J. Tao , S. Chang , Q. Dai , Y. Xiao , Y. Cui , Y. Wang , S. Yu , G. Zheng , S. Zhang , Laser Photonics Rev. 2020, 14, 2000032.

[24] M. Khorasaninejad , W. T. Chen , A. Y. Zhu , J. Oh , R. C. Devlin , D. Rousso , F. Capasso , Nano Lett. 2016, 16, 4595. 27267137 10.1021/acs.nanolett.6b01897

[25] S. Wei , G. Cao , H. Lin , X. Yuan , M. Somekh , B. Jia , ACS Nano 2021, 15, 4769. 33593050 10.1021/acsnano.0c09395

[26] M. Y. Shalaginov , S. An , Y. Zhang , F. Yang , P. Su , V. Liberman , J. B. Chou , C. M. Roberts , M. Kang , C. Rios , Q. Du , C. Fowler , A. Agarwal , K. A. Richardson , C. Rivero‐Baleine , H. Zhang , J. Hu , T. Gu , Nat. Commun. 2021, 12, 1225. 33619270 10.1038/s41467-021-21440-9 PMC7900249

[27] A. She , S. Zhang , S. Shian , D. R. Clarke , F. Capasso , Sci. Adv. 2018, 4, eaap9957. 29507880 10.1126/sciadv.aap9957 PMC5834009

[28] M. Wang , J. S. Lee , S. Aggarwal , N. Farmakidis , Y. He , T. Cheng , H. Bhaskaran , Adv. Sci. 2023, 10, 2204899. 10.1002/advs.202204899 PMC9951390 36596668

[29] B. Xiong , Y. Liu , Y. Xu , L. Deng , C.‐W. Chen , J.‐N. Wang , R. Peng , Y. Lai , Y. Liu , M. Wang , Science 2023, 379, 294. 36656947 10.1126/science.ade5140

[30] Z. Wang , Y. Yao , W. Pan , H. Zhou , Y. Chen , J. Lin , J. Hao , S. Xiao , Q. He , S. Sun , L. Zhou , Adv. Sci. 2023, 10, 2205499. 10.1002/advs.202205499 PMC9896063 36494100

[31] M. Q. Mehmood , J. Seong , M. A. Naveed , J. Kim , M. Zubair , K. Riaz , Y. Massoud , J. Rho , Adv. Sci. 2022, 9, 2203962. 10.1002/advs.202203962 PMC9762282 36285678

[32] T. Badloe , J. Seong , J. Rho , Nano Lett. 2023, 23, 6958. 37478358 10.1021/acs.nanolett.3c01588

[33] C. Chen , X. Xiao , X. Ye , J. Sun , J. Ji , R. Yu , W. Song , S. Zhu , T. Li , Light Sci. Appl. 2023, 12, 288. 38044390 10.1038/s41377-023-01337-6 PMC10694149

[34] C. Li , J. Jang , T. Badloe , T. Yang , J. Kim , J. Kim , M. Nguyen , S. A. Maier , J. Rho , H. Ren , I. Aharonovich , eLight 2023, 3, 19.

[35] S Sarkar , Principles of Light Microscopy: From Basic to Advanced, Springer International Publishing, Cham 2022, pp. 17–56.

[36] Q. Li , I. Ledoux‐Rak , N. D. Lai , Adv. Device Mater. 2015, 1, 4.

[37] S. Zhang , C. L. Wong , S. Zeng , R. Bi , K. Tai , K. Dholakia , M. Olivo , Nanophotonics 2020, 10, 259.

[38] H. S. Khaliq , A. Nauman , J.‐W. Lee , H.‐R. Kim , Adv. Opt. Mater. 2023, 11, 2300644.

[39] J. C. Zhang , M. K. Chen , Y. Liang , X. Hong , M. Wang , Y. Cheng , X. Liu , D. P. Tsai , S. W. Pang , Adv. Funct. Mater. 2023, 33, 2306422.

[40] T. Sun , X. Yang , F. Xu , C. Wang , Nanophotonics 2023, 12, 3243. 39634147 10.1515/nanoph-2023-0142 PMC11501339

