# Supplementary material for: Nonclassical Spin‐Multiplexing Metasurfaces Enabled Multifunctional Meta‐Scope
Source: Small. 2024 Sep 23;20(49):2404003. doi: 10.1002/smll.202404003 (PMC11618737; doi:10.1002/smll.202404003)
Supplement: Supplementary file 1 — Supporting Information [file SMLL-20-2404003-s001.docx]

Supporting material: Nonclassical spin-multiplexing metasurface enabled tri-channel meta-scopes for zoom imaging and simultaneously chiral and achiral imaging

Chuang Sun, Zixuan Wang, Kian Shen Kiang, Oleksandr Buchnev, Dawei Tang, Jize Yan* and Jun-Yu Ou*

Chuang Sun, Zixuan Wang, Kian Shen Kiang, Jize Yan*

School of Electronics and Computer Science, University of Southampton, Southampton SO17 1BJ, UK
E-mail: J.Yan@soton.ac.uk

Oleksandr Buchnev

Optoelectronics Research Centre, University of Southampton, Southampton SO17 1BJ, UK

Dawei Tang

Centre for Precision Technologies, University of Huddersfield, Huddersfield HD1 3DH, UK

Jun-Yu Ou*
University of Southampton, School of Physics and Astronomy, Southampton SO17 1BJ, UK
E-mail: bruce.ou@soton.ac.uk

**Section 1 Meta-atom library for non-classical spin-multiplexing metalens**

As shown in **Figure S1b and S1c**, by fixing a period P of 350nm and a height H of 800nm (**Figure S1a**) for achieving two transmissions ($t_{x}$ and $t_{y}$) higher 90%, the dependence of the propagation phase on the width W and length L of the meta-atom is investigated in the simulation (Lumerical FDTD) where the working wavelength is set as 520nm and the PML boundary condition is applied. According to the phase maps in **Figure S1b**, 14 meta-atoms (**Figure S1c**) are selected for realizing 2$\pi$ coverage of the propagation phase $\varphi_{p}$ by fixing a phase delay $\varphi_{d}$ of ${5\pi}/{12}$.


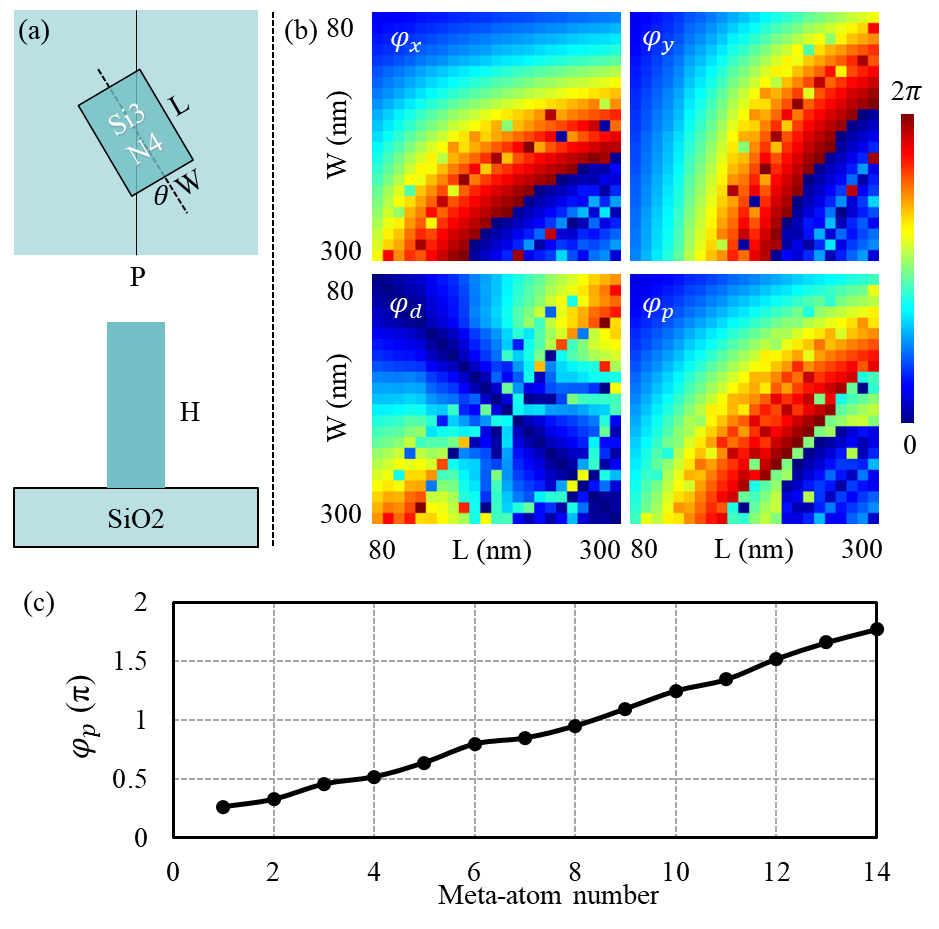


**Figure S1**. Building meta-atoms library. (a) Scheme of the meta-atom; (b) Sweeping width and length for determining phase mapping; (c) The propagation phase of meta-atoms library.

**Section 2 Microscope for characterizing metalens sample**


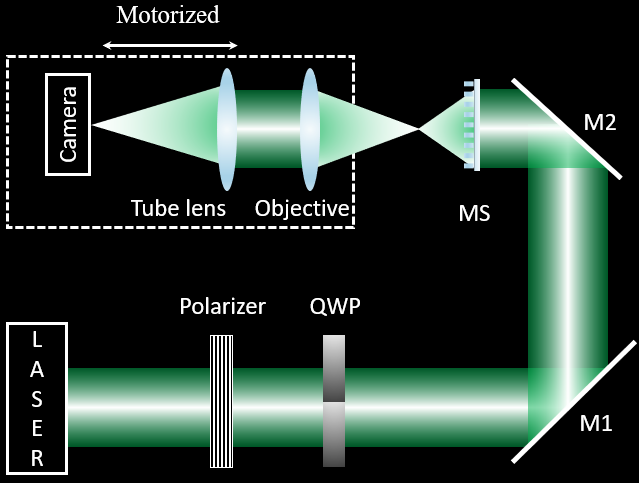


**Figure S2**. Optical configuration for measuring metalens (MS) sample.

As shown in **Figure S2**, a microscope is built in our lab based on a Nikon microscope. A collimated 520 laser is adopted to replace the illumination path and guided to the MS sample via two mirrors (M1 and M2). The focused light via the MS is collected by a 10X objective lens (NA = 0.3) and imaged to a camera via a tube lens with a focal length of 200mm. A polarizer and QWP are placed in the illumination path for controlling the incident light’s polarization.

**Section 3 Fabrication of Si_3_N_4_ metalens**


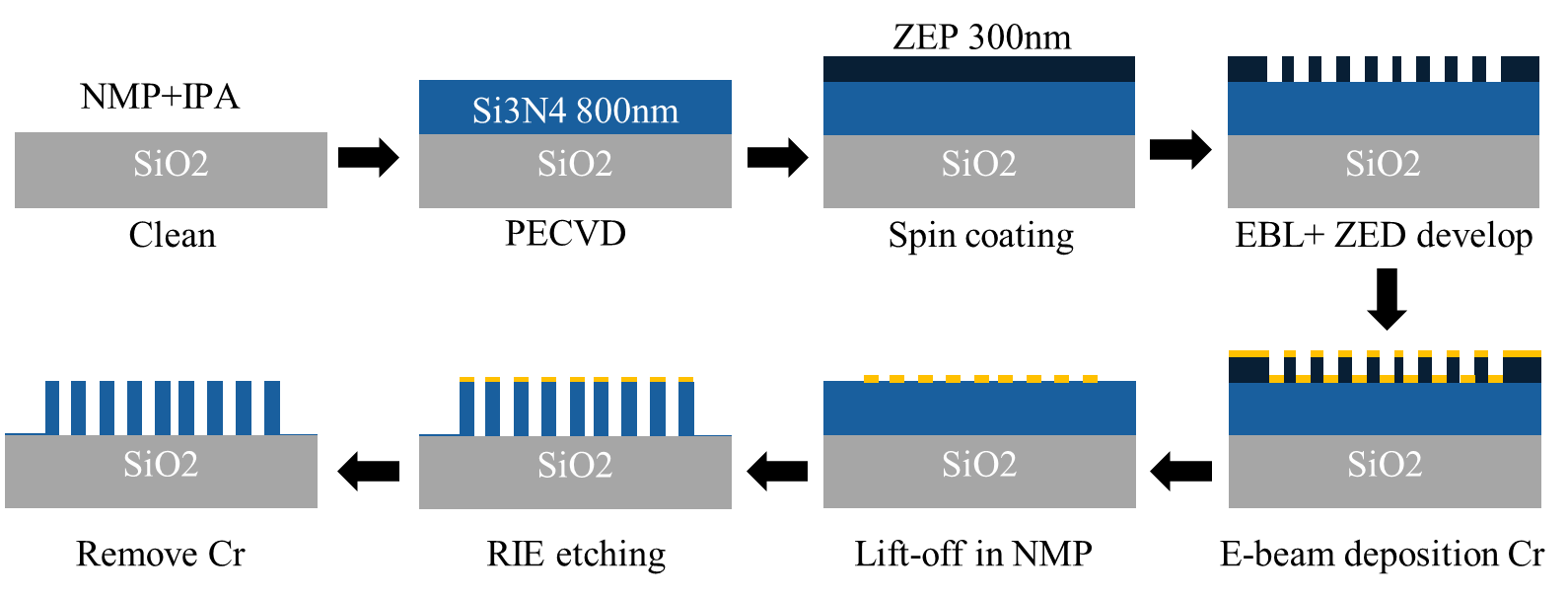


**Figure S3**. Fabrication flow.

The Si_3_N_4_ metalens samples demonstrated in this paper are fabricated in the cleanroom at the University of Southampton following a CMOS-compatible process as shown in **Figure S3**. The metalens samples are fabricated on a double-side polished SiO_2_ wafer. The wafer is first cleaned in NMP for 10 minutes and IPA for 10 minutes. Then, a layer of Si_3_N_4_ (800nm in thickness) (**Figure S4**) is deposited at a rate of 25nm/min via PECVD at 350$℃$. ZEP520A resist is spin coated on the wafer and baked on a hot plate for 3mins at 180$℃$. Subsequently, the metalens pattern is defined by the e-beam lithography (EBL) and development process in ZED solution. As the hard mask, a 35nm-thick Cr layer is coated on the substrate by e-beam evaporation, followed by a lift-off in NMP. Consequently, the designed patterns are transferred to the Cr layer. Then, the wafer with the patterned Cr layer is etched by reactive ion etching (RIE) at a rate of 27nm/min. Finally, metalens samples are obtained after removing Cr layer via Cr etchant.


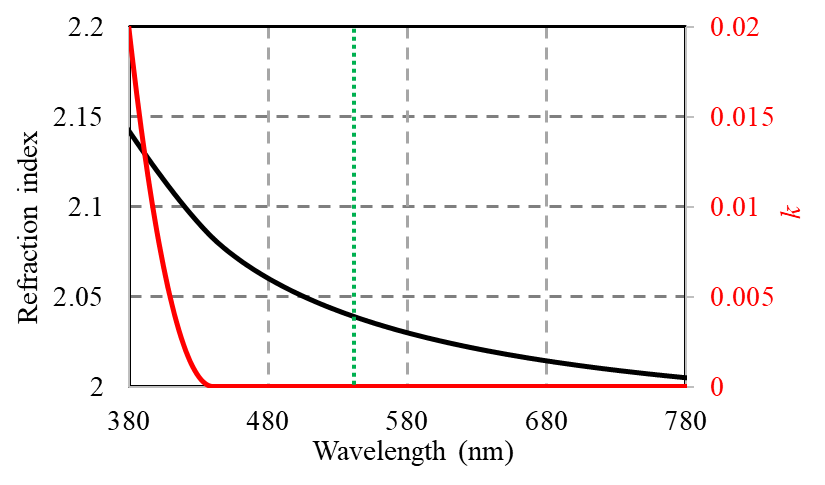


**Figure S4**. Characterization of PECVD Si_3_N_4_ film (800nm).

**Section 4 Design of *q*-metalens**

As the *q*-metalens works as a tube lens in the integrated microscope, the *q*-metalens only needs to focus incident light to a focal point. Therefore, an isotropic meta-atom (H = 800nm, P = 350nm, and W = L) library (**Figure S5**) is built to design the *q*-metalens. As shown in **Figure 5b**, the inclined light needs to be perfectly focused into the focal plane to realize large FOV. Therefore, the quadratic phase profile (**Equation 5**) is adopted to design the *q*-metalens.


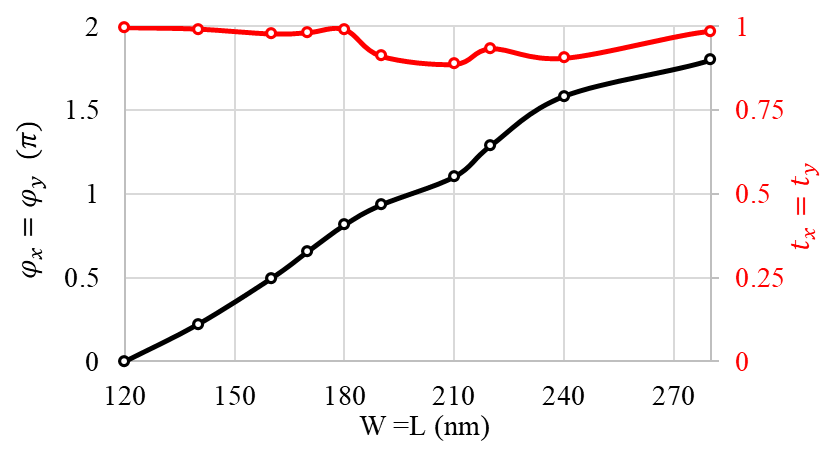


**Figure S5**. Isotropic meta-atom library for *q*-metalens.

**Section 5 Phase profiles of the in-plane tri-foci metalens**

As shown in **Figure S6a**, a hyperbolic phase profile $\Phi_{2}(f=6mm)$ is adopted to realize an on-axis diffraction-limited focal point with an NA of 0.1. Meanwhile, an off-axis hyperbolic phase profile $\Phi_{3}=\Phi_{off}(f=6mm, \beta=10^{\circ})$ (**Figure S6b**) with a tilt angle $\beta=10^{\circ}$ is adopted to realize an off-axis LCP focal point with an NA of 0.1. As a result, a phase profile $\Phi_{1}=2\Phi_{2}-\Phi_{3}$ (**Figure S6c**) is encoded to the RCP channel.

To investigate the working performance of the RCP channel of the in-plane tri-foci metalens, an expected phase profile $\Phi_{1e}=\Phi_{off}(f=6mm, \beta=-10^{\circ})$ is demonstrated in **Figure S6d**, and the unwrapped phase difference $\Phi_{1d}=\Phi_{1}-\Phi_{1e}$ is shown in **Figure S6e and S6f**. As the peak-valley value of phase difference $\Phi_{1d}$ is only 7$\pi$ and much smaller than the expected phase profile $\Phi_{1e}$. The $\Phi_{1}$ can be regarded as an estimated phase profile of the $\Phi_{1e}$. Therefore, we conceive that the RCP channel can achieve a focal point with an NA of 0.1. In addition, the phase difference $\Phi_{1d}$ varying along the Y direction (**Figure S6e and S6f**) would lead to a non-perfect focus on the Y direction of the RCP channel in the experiment.


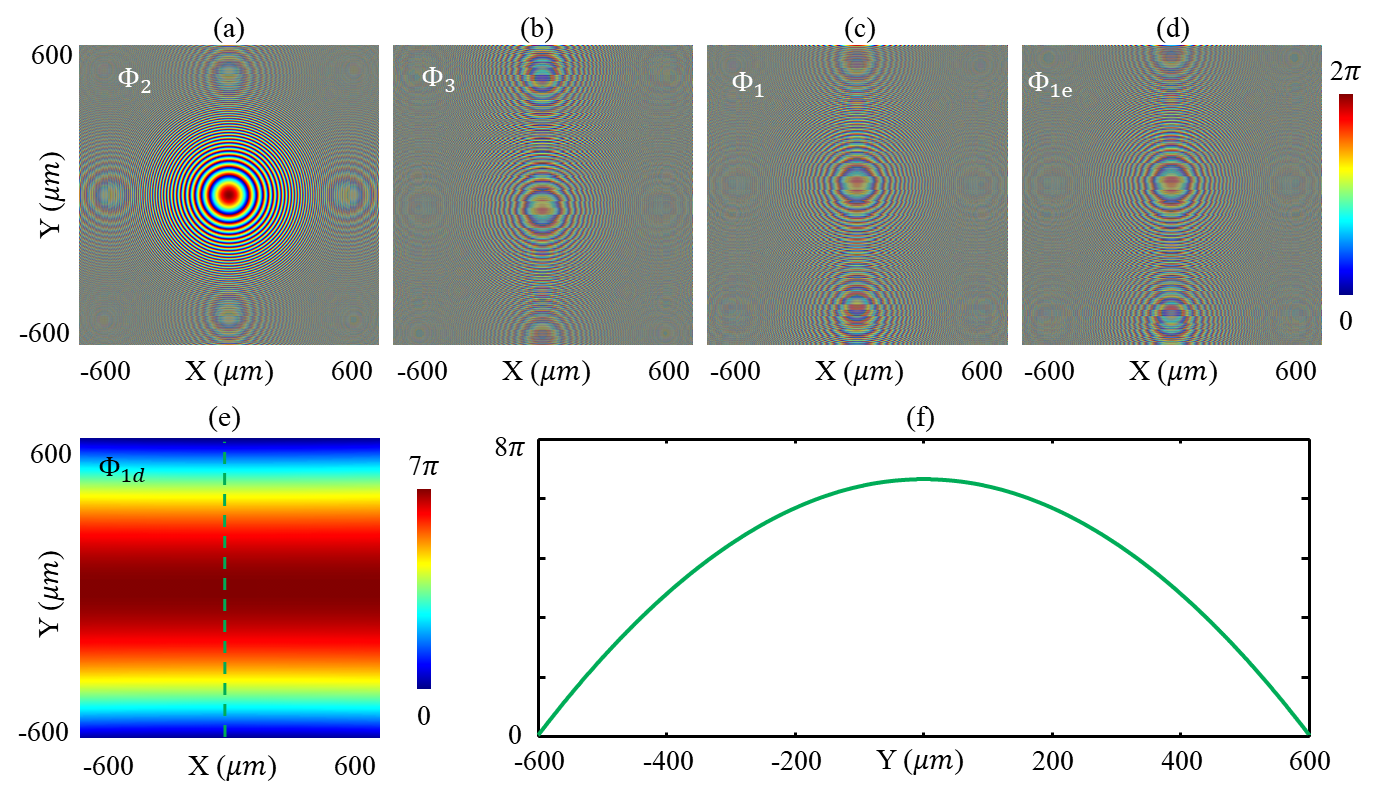


**Figure S6.** (a) – (c) are the phase profiles $\Phi_{2}(f=6mm)$, $\Phi_{3}(f=6mm, \beta=10^{\circ})$, and $\Phi_{1}=2\Phi_{2}-\Phi_{3}$ encoded on the metalens sample, (d) is an expected tilt focusing phase profile $\Phi_{1e}=\Phi_{off}(f=6mm, \beta=-10^{\circ})$ of the RCP channel, (e) shows the phase difference $\Phi_{1d}=\Phi_{1}-\Phi_{1e}$, (f) is the 1D phase profile of the $\Phi_{1d}(X=0)$.

**Section 6 Magnification and resolution of the simultaneously chiral and achiral meta-scope system**

A singlet meta-scope system is built for simultaneously achiral and chiral imaging based on the in-plane tri-foci metalens sample as shown in **Figure S7**. The imaged object is illuminated by a focused 520nm laser beam via the lens L1, and a polarizer and QWP are placed to control the laser beam’s polarization state. Then, the scattered light from the object is imaged on a whiteboard (WB) by the in-plane tri-foci metalens sample. In addition, a ruler is placed in front of the WB to calibrate the size of the achiral and chiral images as well as the magnification of the microscope system (**Figure S7b and S7c**). According to the real dimension of the USAF 1951 image in Figure S6c, the meta-scope has a magnification of 53 times. As shown in **Figure S7d**, the meta-scope can resolve element 3 in group 7, which illustrates the microscope possessing a diffraction-limited resolution of 3.1$\mu m$.


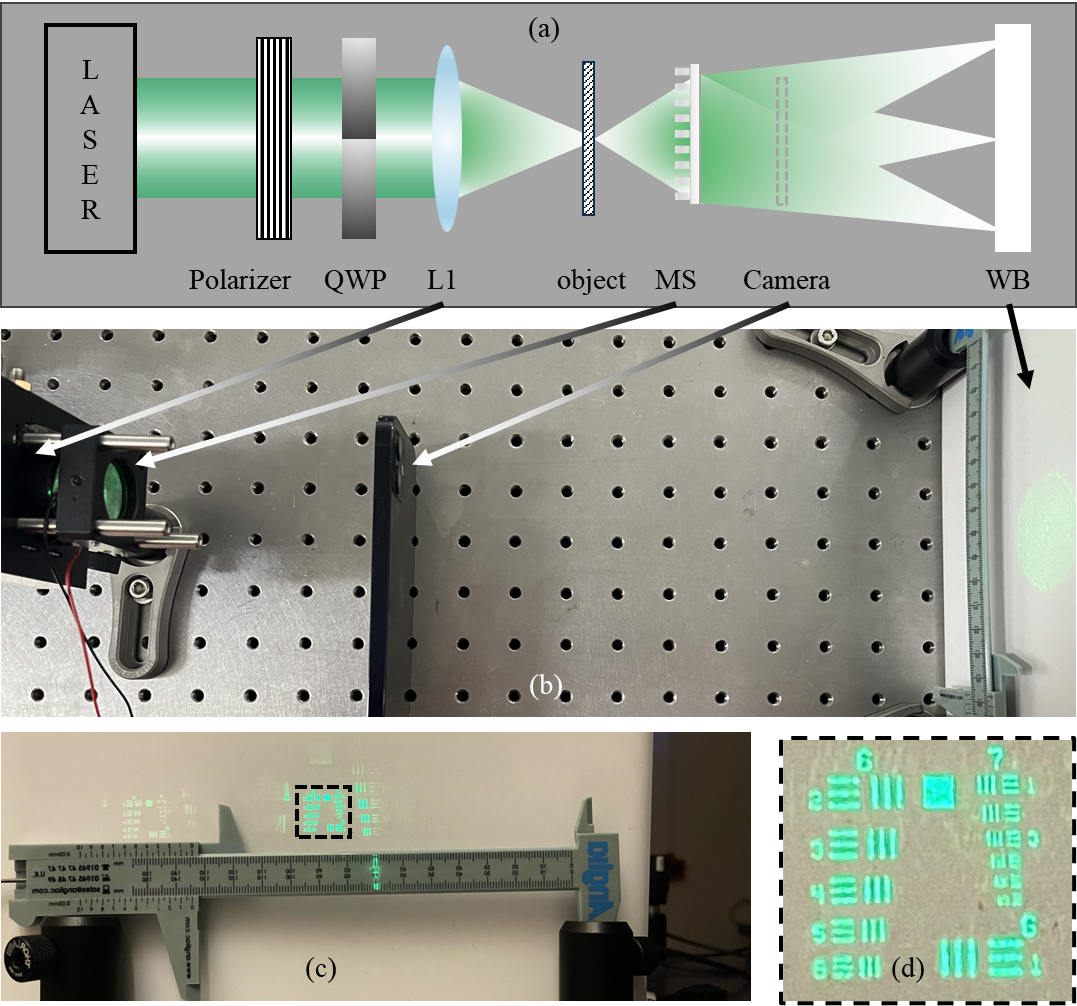


**Figure S7**. (a) Optical configuration of the metalens-based microscope for achiral and chiral imaging, (b) the experiment setup, (c) the initial picture captured by the iPhone camera, (d) the zoom-in image of the marked region in (c) which shows the imaging resolution of 3.1$\mu m$.

**Section 7 Liquid crystal cell fabrication**

A liquid crystal cell was fabricated from two indium oxide (ITO) coated glass substrate. A polyimide layer was spin-coated on the top of the ITO substrate and rubbed with a velvet cloth for planar liquid crystal alignment. The substrates were separated by 3 um SiO2 ball spacers, and the gap was filled with E7 LC using a vacuum filling station.

Half-waveplate and quarter-waveplate voltages were identified by placing a cell between crossed linear polarizers at 45$^{\circ}$ with respect to polarizers axis and measuring laser beam transmittance depending on the voltage applied to ITO electrodes. The half-waveplate condition corresponds to a maximum in transmittance intensity, while the quarter-waveplate condition corresponds to half of the maximal intensity, respectively.
